# Transient but not chronic hyperglycemia accelerates ocular glymphatic transport

**DOI:** 10.1186/s12987-024-00524-w

**Published:** 2024-03-12

**Authors:** Christine Delle, Xiaowei Wang, Michael Giannetto, Evan Newbold, Weiguo Peng, Ryszard Stefan Gomolka, Antonio Ladrón-de-Guevara, Neža Cankar, Elise Schiøler Nielsen, Celia Kjaerby, Pia Weikop, Yuki Mori, Maiken Nedergaard

**Affiliations:** 1 Center for Translational Neuromedicine, Faculty of Medical and Health Sciences, University of Copenhagen, Blegdamsvej 3B, 2200 Copenhagen N, Denmark; 2 Center for Translational Neuromedicine, University of Rochester Medical School, Elmwood Avenue 601, 14642 Rochester, NY USA; 3 School of Medicine, University of California, San Francisco, 10 Koret Way, 94117 San Francisco, CA USA

**Keywords:** Perivascular spaces, Ocular glymphatic system, Diabetes, Cerebrospinal fluid, Retina, Retinal ganglion cells, Glial lamina, electron microscopy, Magnetic resonance imaging

## Abstract

Glymphatic transport is vital for the physiological homeostasis of the retina and optic nerve. Pathological alterations of ocular glymphatic fluid transport and enlarged perivascular spaces have been described in glaucomatous mice. It remains to be established how diabetic retinopathy, which impairs vision in about 50% of diabetes patients, impacts ocular glymphatic fluid transport. Here, we examined ocular glymphatic transport in chronic hyperglycemic diabetic mice as well as in healthy mice experiencing a daily transient increase in blood glucose. Mice suffering from severe diabetes for two and four months, induced by streptozotocin, exhibited no alterations in ocular glymphatic fluid transport in the optic nerve compared to age-matched, non-diabetic controls. In contrast, transient increases in blood glucose induced by repeated daily glucose injections in healthy, awake, non-diabetic mice accelerated antero- and retrograde ocular glymphatic transport. Structural analysis showed enlarged perivascular spaces in the optic nerves of glucose-treated mice, which were absent in diabetic mice. Thus, transient repeated hyperglycemic events, but not constant hyperglycemia, ultimately enlarge perivascular spaces in the murine optic nerve. These findings indicate that fluid transport in the mouse eye is vulnerable to fluctuating glycemic levels rather than constant hyperglycemia, suggesting that poor glycemic control drives glymphatic malfunction and perivascular enlargement in the optic nerve.

**Supplementary Information:**

The online version contains supplementary material available at 10.1186/s12987-024-00524-w.

## Background

Diabetes type I and II are both associated with an increased risk for Alzheimer’s [1, 2] and small vessel disease [3, 4]. Diabetes injures the microvasculature [5], and tissues with high microvascular density, such as the retina or kidney, are particularly vulnerable. Diabetic retinopathy is recognized as a leading cause of visual impairment and blindness. Diabetic retinopathy is characterized by vascular pathologies which include increased capillary permeability, local occlusions, ischemia, microaneurysms, and neovascularization in the proliferative stage of the disease [6, 7].

The highly metabolically active cells of the neuroretina require an interstitial fluid transport system to clear metabolic waste and excess water. The recently described ocular glymphatic system performs just such a function by subserving the transport of fluid and metabolites from the retina into the optic nerve [8]. Wang et al. demonstrated the transport of intravitreally injected tracers in mice via retinal ganglion cell (RGC) axons across the glial lamina [8], the analogue to the human lamina cribrosa. This study showed initial accumulation of tracers in the perivascular space surrounding the central retinal vein, followed by export along meningeal lymphatic vessels located in the dura surrounding the optic nerve [8]. Conversely, tracers introduced into the cerebrospinal fluid (CSF) via cisterna magna (CM) infusion enter the optic nerve along periarterial spaces [8, 9]. The term “ocular glymphatic pathway” is commonly applied to this uniquely bidirectional tracer transport in the optic nerve [8–10]. To distinguish the elements of bidirectional transport, we designate the movement of intravitreally injected tracer away from the eye as anterograde ocular glymphatic transport, as distinct from the retrograde ocular glymphatic transport of intracisternal administered CSF tracers towards the eye [11]. Ocular glymphatic transport, similarly to the brain glymphatic system [12], depends on astrocytic expression of the water channel aquaporin-4 (AQP4) [8]. Furthermore, we note that anterograde glymphatic transport carries across the glial lamina only solutes such as amyloid-β that are taken up by retinal ganglion neurons and transported within their axonal projections. Amyloid-β is then released from the axons, after passing the glial lamina and initially accumulates along the central retinal vein followed by clearance via dural lymphatic vessels. In contrast, CSF tracers injected into cisterna magna enter the optic nerve via retrograde transport along the periarterial spaces [8, 11]. All published studies on ocular glymphatic transport have concluded that CSF tracers never enter inner ocular structures, including the vitreous body or the retina [8, 9, 11, 13], CSF tracers enter the optic nerve along the periarterial spaces but exit along dural lymphatic vessels prior to reaching the glia lamina [11]. It is in this regard crucial to examine tracer distribution along the entire length of the optic nerve, rather than in individual segments on sectioned tissue as tracer distribution within the optic nerve exhibit intensity variation as a function of the distance from the point of tracer entry. The sectioned optic nerve tissue lacks precise positional information which can lead to misleading results and interpretations [10, 13].

Hyperglycemia is believed to be central in the pathogenesis of diabetic retinopathy [6, 14–16]. If left poorly controlled, fluctuating blood glucose levels in diabetic individuals lead to accelerated vascular damage and increase oxidative stress [17, 18]. As diabetes can severely impact retinal and optic nerve vasculature, it follows that diabetes might also affect glymphatic fluid transport in the optic nerve. While magnetic resonance imaging (MRI) studies in rats have pointed towards brain glymphatic alterations in diabetes [19, 20], there are no corresponding investigations of the ocular glymphatic system despite the clinical importance of diabetic retinopathy. Thus, we tested the hypothesis that ocular glymphatic transport is impaired in a mouse streptozotocin (STZ) model of type I diabetes. Contrary to our hypothesis, chronic and severely diabetic mice exhibited no sign of perturbed transport compared to healthy controls.

Diabetic retinopathy can arise despite yearlong insulin treatment and is facilitated by fluctuations of glucose levels [14, 21–24]. Indeed, hyperglycemia in diabetic patients can elevate blood osmolality [25, 26] which, in uncontrolled hyperglycemic individuals, can lead to a hyperosmolar hyperglycemic syndrome [27, 28]. It is known that acute plasma hypertonicity increases brain glymphatic influx [29]. Thus, we further hypothesized that the oscillating blood glucose levels, rather than constant blood glucose elevation, are more detrimental to ocular glymphatic transport. To test this hypothesis, we quantified ocular glymphatic transport in healthy mice treated with daily intraperitoneal high doses of glucose, as compared to saline-treated controls.

## Materials and methods

### Animals

#### Diabetes induction

CD1 mice (Janvier-Labs, France) of either sex were housed under standard housing conditions in temperature-controlled rooms at 21 °C and a 12/12-hour light/dark cycle (lights on 6 AM), and water and food *ad libitum*. At the age of six weeks unanesthetized mice were injected intraperitoneally (i.p.) with a single high dose (150 mg/kg) of streptozotocin (STZ) (MP Biomedicals (ICN10055701) or Selleckchem (S1312)) dissolved in Na-Citrate buffer (0.1 M, pH 4.5). Control animals were injected with buffer only. We confirmed a hyperglycemic state at 48 h after STZ administration; we excluded from the study any mice not manifesting hyperglycemia (see supplementary methods). Animals were randomly assigned to the conducted experiments.

#### Repeated glucose challenge

Healthy unanaesthetised male 8-week-old CD1 mice were i.p. injected daily (except weekends) with a glucose solution (20 µL/g bodyweight, 1 M glucose solution in sterile isotonic saline, thus 3.6 g glucose/kg body weight), or with equivalent volumes of sterile isotonic saline solution for one month. To simplify the procedure, mice of one cage received the same injection volume calculated according to the weekly average body weight per cage. Mice underwent glymphatic tracer injections at 72 to 120 h after receiving the last glucose or saline injection. Blood glucose values were measured prior to the final experiments as described in supplementary methods. All experiments carried out at the University of Copenhagen were approved by the Animal Experiments Council under the Danish Ministry of Environment and Food (license number: 2015-15-0201-00664), in accordance with the European directive 2010/63/EU. Experiments conducted at the University of Rochester Medical Center were approved by the University of Rochester Committee on Animal Resources.

### Glymphatic experiments

Glymphatic tracer injections were administered to deeply anesthetized animals [ketamine/xylazine anesthesia (100 mg/kg and 20 mg/kg i.p., respectively)] at two or four months after diabetes induction. Experiments were conducted during the inactive period of the animals between 7 AM– 6 PM.

**Intravitreal tracer injection** was performed as previously described [8]. At a flow rate of 0.2 µL/min, we infused a mixture containing 1 µL of 0.5% HiLyte Fluor 488-conjugated hAβ (Human Beta-Amyloid (1–40) HiLyte Fluor 488-labeled, AS-60491-01, AnaSpec Inc., USA) diluted in artificial cerebral spinal fluid (aCSF) (155 mM NaCl, 3.5 mM KCl, 1 mM CaCl_2_, 1 mM MgCl_2_, and 2 mM NaH_2_PO_4_, pH 7.4, 300 mOsm). During tracer circulation under anesthesia, we anointed the cornea with eye lubricant (Aptus^®^ SentrX Eye Gel, Orion Pharma A/S, Finland). In a subset of mice, we tested glial lamina integrity by intravitreally injecting a low molecular weight dextran (Dextran 3 kDa, D3308, ThermoFisher Scientific), as previously described [8].

**Intracisternal tracer injection** was performed as previously described [8]. The diabetic experimental group was infused in cisterna magna with 15 µl of 10 kDa Dextran-AlexaFluor™ 555 (D34679, ThermoFisher Scientific) at a concentration of 0.5% (w/v, in aCSF) at a rate of 1.5 µL/min over a period of 10 min. For glucose-treated mice, 10 µl bovine serum albumin (BSA) Alexa Fluor™ 647 conjugate (A34785, 66 kDa, ThermoFisher Scientific) at 0.5% concentration (w/v, in aCSF) was infused at a rate of 2 µL/min over a period of 5 min. Notably, previous analysis showed that CSF tracers of the present range of molecular weight (1–80 kDa) do not impact glymphatic flow [30]. However, intracisternal injection at this volume and rate will briefly elevate intracranial pressure (2.5 mmHg, 5 min), which falls within the physiological range of intracranial pressure fluctuations and is without effect on glymphatic tracer distribution [30, 31]. Intracisternal tracer infusions started at the same time as intravitreal injections. All mice were sacrificed after 30 min of tracer circulation and the optic nerves harvested [33, 34]. Of note, studies of retrograde ocular glymphatic transport entail tracer injection in cisterna magna, with transport in the subarachnoid space before reaching the optic nerve. Anatomical differences of the subarachnoid space and/or the velocity of CSF transport may thus contribute to the inter-animal variability of retrograde ocular glymphatic transport [8, 11].

A subset of mice underwent vascular labelling with lectin (see supplementary methods). The freshly harvested optic nerves were immediately imaged using a macroscope. Of note, acute macroscopic imaging of fresh tissue does not distinguish tracer signal within the dura and the optic nerve but avoids tracer artefacts in fixed tissue [32]. Fluorescent light intensity and exposure times were kept constant for each fluorophore for all imaged nerves, and fluorescence analysis was conducted as previously described [8].

### Tissue fixation, preparation, and imaging

Optic nerves and eyeballs were collected and fixed by immersion in 4% paraformaldehyde for 1 and 2 h, respectively, at room temperature. To assess hAβ tracer distribution in retinas, retina whole mounts were imaged using a standard fluorescence microscope (Nikon ECLIPSE Ni-E) equipped with a digital camera (Mono-Camera Nikon DS-Fi3) and controlled by imaging software (NIS-Elements Imaging software AR 4.60.00). Images were analyzed using ImageJ (version 2.1.0/1.53c), as described in supplementary methods.

For examining glial lamina integrity, enucleated eyes were passed through a sucrose-PBS concentration series ending with 30% sucrose. The optic nerve around the glial lamina region was cut into serial 20 μm cross sections using a cryostat (Leica CM1950). Tissue sections were then imaged using a confocal microscope. In addition, we conducted electron microscopy of the glial lamina as described in supplementary methods.

To examine optic nerve vasculature and perivascular spaces (PVS) of lectin-perfused mice, we prepared 25 μm-thick serial cryostat cross-sections (as above). Confocal images were analyzed using ImageJ (version 2.1.0/1.53c). The lectin-positive area was quantified to indicate total vascularization, and vessel length was measured. For calculating PVS volume fraction, the vessel diameter (lectin signal) was subtracted from the area containing tracer signal labelling of the PVS surrounding the blood vessels. To correct for potential effects of vessel sizes, we also calculated the PVS ratio by dividing the diameter of the tracer signal with the diameter of the corresponding lectin signal.

### MRI

Animals were imaged two or four months after STZ or sham injection. Mice were anesthetized with isoflurane (3% induction, 1-1.5% maintenance) in a 1/1 mixture of air/oxygen. The body temperature was maintained at 37 ± 0.5 °C with a thermostatically controlled waterbed, and was monitored, along with the respiratory rate, using an MR-compatible small animal remote monitoring system (SA Instruments, NY, USA). MRI was performed in a 9.4 T animal scanner (BioSpec 94/30 USR, Paravision 6.0.1 software, Bruker BioSpin, Ettlingen, Germany) with a ^1^H cryogenically-cooled quadrature-resonator Tx/Rx coil (CryoProbe) and 240 mT/m gradient coil (BGA-12 S, Bruker). CSF volumetry was conducted using 3D constructive interference steady-state (3D-CISS). Every 3D-CISS image was calculated as a maximum intensity projection from 4 realigned 3D-TrueFISP volumes with 4 orthogonal phase encoding directions (TR/TE 3.9/1.95 ms, FA 50º, FOV 19.2 × 15 × 16 mm, matrix 246 × 192 × 205). The N4 bias field was corrected using Advanced Normalization Tools (ANTs N4 bias correction) [33]. The 3D-CISS volume was reformatted with curved planar reformation (CPR) using Horos (v.4.0.0 RC1; The Horos Project, MD, USA). Afterwards, the optic nerve volume was semi-automatically segmented by using region growing with ITK-snap (version 3.8.0) [34]. The occupation rates of the optic nerve and the subarachnoid space (SAS) were simply calculated as (Volume_SAS / Volume_totalCanalVolume) × 100 or (Volume_opticNerve / Volume_totalCanalVolume) × 100, respectively.

### Statistical analysis

Data are presented as mean ± SD using GraphPad Prism 9 (GraphPad Software). Shapiro-Wilk test was utilized to determine the normality of the data. For comparisons of means between the two groups, a two-tailed Welch *t* test was performed. For comparison of data sets with more than two groups one-way ANOVA with Geisser-Greenhouse correction or two-way ANOVA with Tukey’s modification (non-repeated) or Šidák modification (repeated measures) was applied. *P* values indicated as follows: *P ≤ 0.05, **P ≤ 0.01, ***P ≤ 0.005, and ****P ≤ 0.001.

## Results

### Ocular glymphatic fluid transport remains unaltered in hyperglycemic diabetic mice

To explore the impact of diabetic retinopathy on ocular glymphatic transport, mice were studied two and four months after the STZ injection. Administration of a single high dose of STZ in mice depletes insulin producing β-cells of the pancreas, thus rapidly leading to a constant hyperglycemic state [35]. Observations of chronic hyperglycemia (> 25 mmol/L), polyuria, reduced weight gain, and reduced activity during the awake phase all confirmed the development of a type I diabetic phenotype (Supp. Figure 1). Awake mice trained to tolerate intraocular pressure (IOP) measurements showed an early IOP elevation that declined transiently around 3.5 weeks following the initial injection and remained elevated from 4.5 weeks onwards (Suppl. Figure 2 A). Of note, there were no differences in the IOP values in control and diabetic mice when anesthetized just prior to intravitreal tracer injections (Suppl. Figure 2B).

**Fig. 1:**
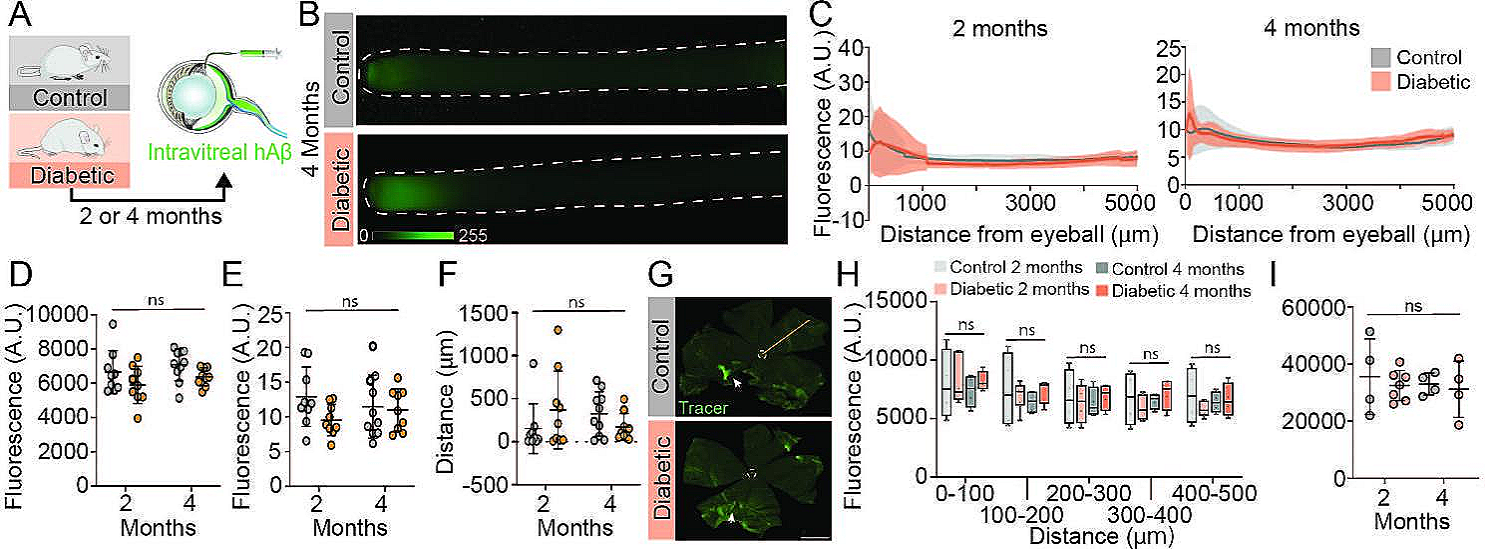
Ocular glymphatic clearance is unaltered in diabetic CD1 mice. (A) Schematic diagram of experimental approach. (B) Representative macroscopic images of optic nerves from control and diabetic mice four months after sham or STZ injection. (C) Total tracer signal over the entire length of the optic nerve at two and four months after onset of STZ-induced diabetes, with subtraction of background fluorescence of the contralateral non-injected control optic nerve (n = 9–10). (D) Plot of total tracer signal (arbitrary units (A.U.)), (E) peak signal intensity (A.U.), and (F) peak signal travel distance (µm). n = 9–10, ns = P > 0.05 between indicated groups by two-way ANOVA with Tukey’s correction (D-F). (G) Representative retina wholemounts imaged by epifluorescent microscopy for quantitation of tracer distribution. White circle– retina center, white arrow– site of intravitreal injection, orange line– example of drawn line ROI. Scale bar 1 mm. (H) Total tracer signal (A.U.) in control and diabetic retinas of the two- and four-month time points for different segments of the retina (distance ascending from retina center in µm). (I) Total tracer signal (A.U.) for whole retinas of two- and four-month diabetic and control mice. H + I) n = 4–7, ns = P > 0.05 between indicated groups by two-way ANOVA with Tukey’s correction. All graphs show mean ± SD

There is no existing methodology for dynamically imaging anterograde ocular glymphatic transport in live mice, as invasive surgical access to the optic nerve would interfere with fluid transport [36]. We here analyzed anterograde glymphatic flow ex vivo following intravitreal injection of HiLyte Fluor-488-tagged hAβ tracer as previously described [8] (Fig. 1A). The freshly resected optic nerves harvested after 30 min of tracer circulation showed no difference in total detected tracer signal between the diabetic and the control mice (Fig. 1B). The peak signal intensity and peak signal distance were also of similar magnitude between the two groups (Fig. 1C-G). Tracer distribution in extracted retinas likewise revealed no difference in tracer signal between groups (Fig. 1H-I). Histological examination revealed loss of retinal ganglion cells, retinal vascular changes, and leaky blood vessels in the STZ-treated group (Suppl. Figure 3), documenting that all were severely diabetic.

**Fig. 2:**
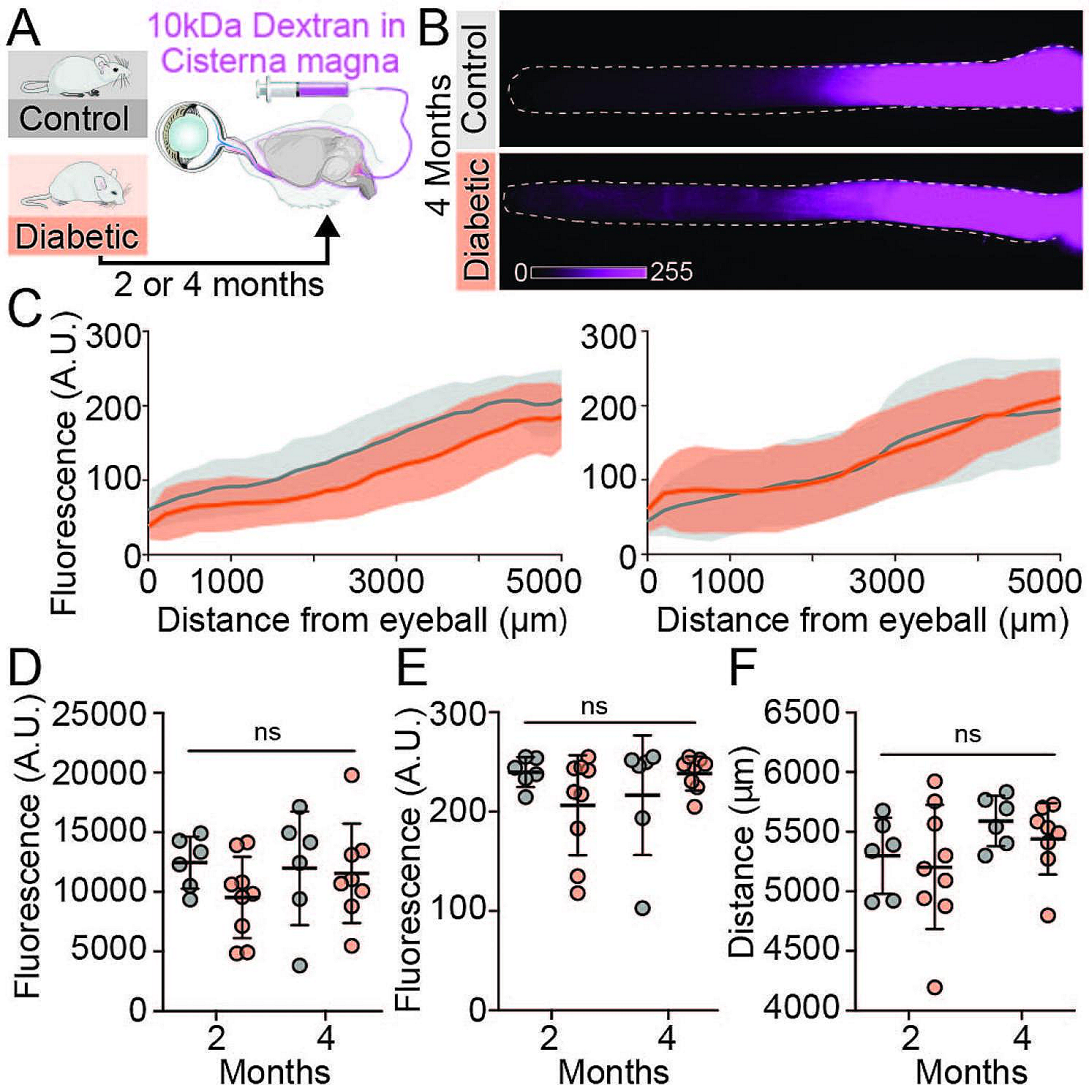
Ocular glymphatic CSF influx along the optic nerve remains unchanged in diabetic mice. (A) Experimental design of CSF tracer distribution in the optic nerve after intracisternal tracer injection. (B) Representative macroscopic images of CSF tracer distribution along optic nerves of four-month diabetic or control mice. (C) Distribution of CM-injected tracer depicting glymphatic CSF influx along the entire optic nerves (distance ascending from anterior to posterior) Left: two-months, right: four-month time point after onset of STZ diabetes (n = 6–9). (D) Total CM tracer signal in the optic nerve, (E) peak signal intensity and (F) peak signal distance travelled. Distance ascending from eyeball. n = 6–9, ns = P > 0.05 between indicated groups by two-way ANOVA with Tukey’s correction. All graphs show mean ± SD

To investigate retrograde ocular glymphatic transport along the optic nerve, a CSF tracer (10 kDa dextran) was administered intracisternally (Fig. 2) concurrently with intravitreal injection described above (Fig. 1). There were no group differences of 10 kDa dextran CSF tracer distribution in diabetic optic nerves with respect to total tracer signal, peak signal intensity, or peak signal distance (Fig. 2D-F). These findings indicate that retrograde ocular glymphatic transport is unaffected after two and four months of severe diabetes.

Notably, the lymphatic vessels in meninges surrounding the optic nerve did neither exhibit structural changes in the diabetic mice (Suppl. Figure 5; mean lymph vessel diameter and the signal intensity for the lymphatic vessels marked LYVE-1 were likewise similar between groups).

### The glial lamina integrity is preserved in diabetic mice

**Fig. 3:**
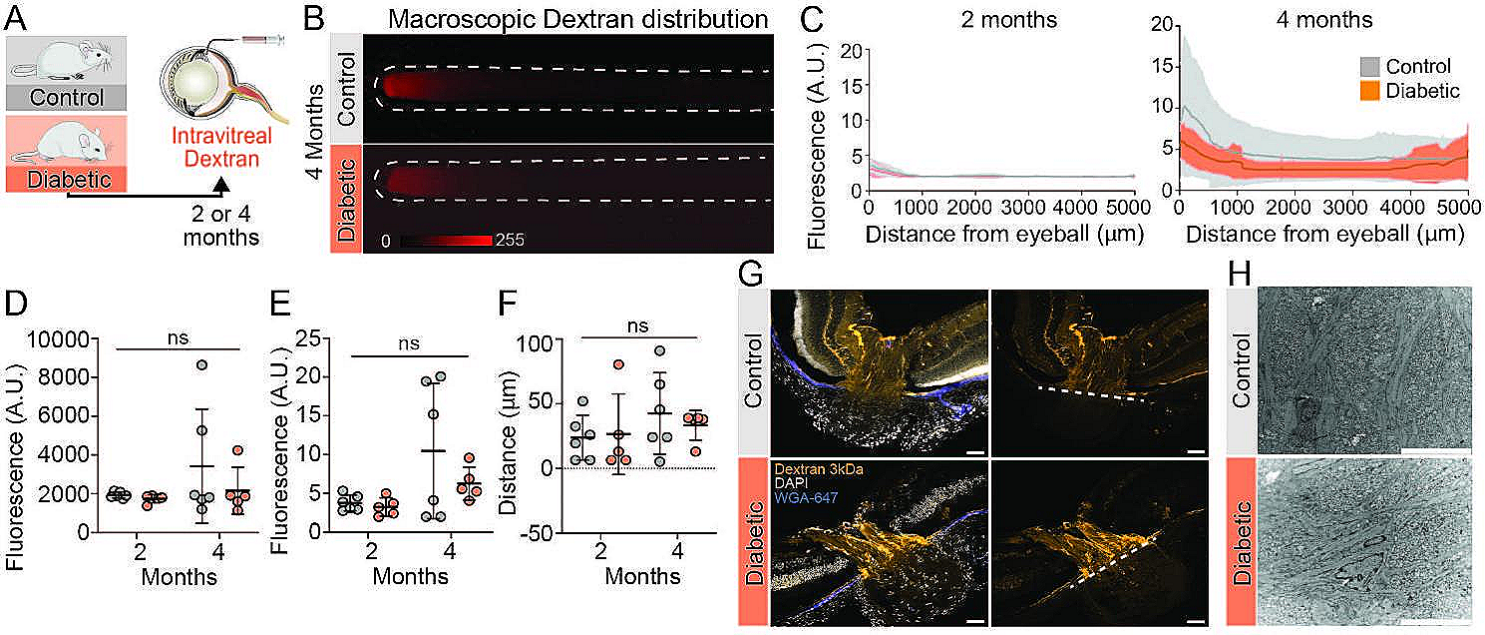
Glial lamina integrity remains unaltered in diabetic mice. (A) Schematic diagram illustrating the experimental approach to evaluate the integrity of the glial lamina using intravitreally administered dextran tracer. (B) Representative macroscopic images of optic nerves after dextran tracer injection into the vitreous humor. The image brightness was increased to show the optic nerve outlined by the high autofluorescence signal. (C) Total tracer signal over the entire length of the optic nerve at two (left) and four (right) months after onset of diabetes (n = 5–6). (D) Plot of total fluorescent tracer signal (A.U.), (E) peak signal intensity and (F) peak signal travel distance. Distance ascending from the eyeball. N = 5–6, ns = P > 0.05 between indicated groups by two-way ANOVA with Tukey’s correction (D-F). (G) Representative confocal images of retina cross sections showing the optic nerve head region and dextran tracer (3 kDa) distribution. White dashed line indicates glial lamina region. Scale bar 50 μm. (H) Standard TEM images of glial lamina in cross section. Scale bar 10 μm, n = 2. All graphs show mean ± SD

To analyze whether diabetes compromised the barrier function of the glial lamina, a dextran (3 kDa) tracer was next administered intravitreally. Intravitreally administered dextran tracers are not taken up by RGC and can therefore not cross the glial lamina [8, 11]. We have earlier observed an excessive transport of dextran across the glial lamina barrier in two murine models of glaucoma in conjunction with structural defects in the glial lamina [8], However, no enhancement of transport across the glial lamina was observed in the diabetic mice or age-matched controls (Fig. 3). Total tracer signal was low and comparable between both groups (Fig. 3D). Also, the peak intensity and peak signal travelled in the optic nerve were comparably low (Fig. 3E, F). Additional confocal imaging of injected eyeballs confirmed the absence of dextran tracer transport over the glial lamina into the optic nerve in both groups (Fig. 3G). Moreover, electron microscopy confirmed the morphological integrity of the glial lamina after four months of diabetes (Fig. 3H). Thus, we did not detect functional or structural disturbances of the glial lamina in chronic diabetic mice.

### In vivo CSF space reduction in diabetic optic nerves

**Fig. 4:**
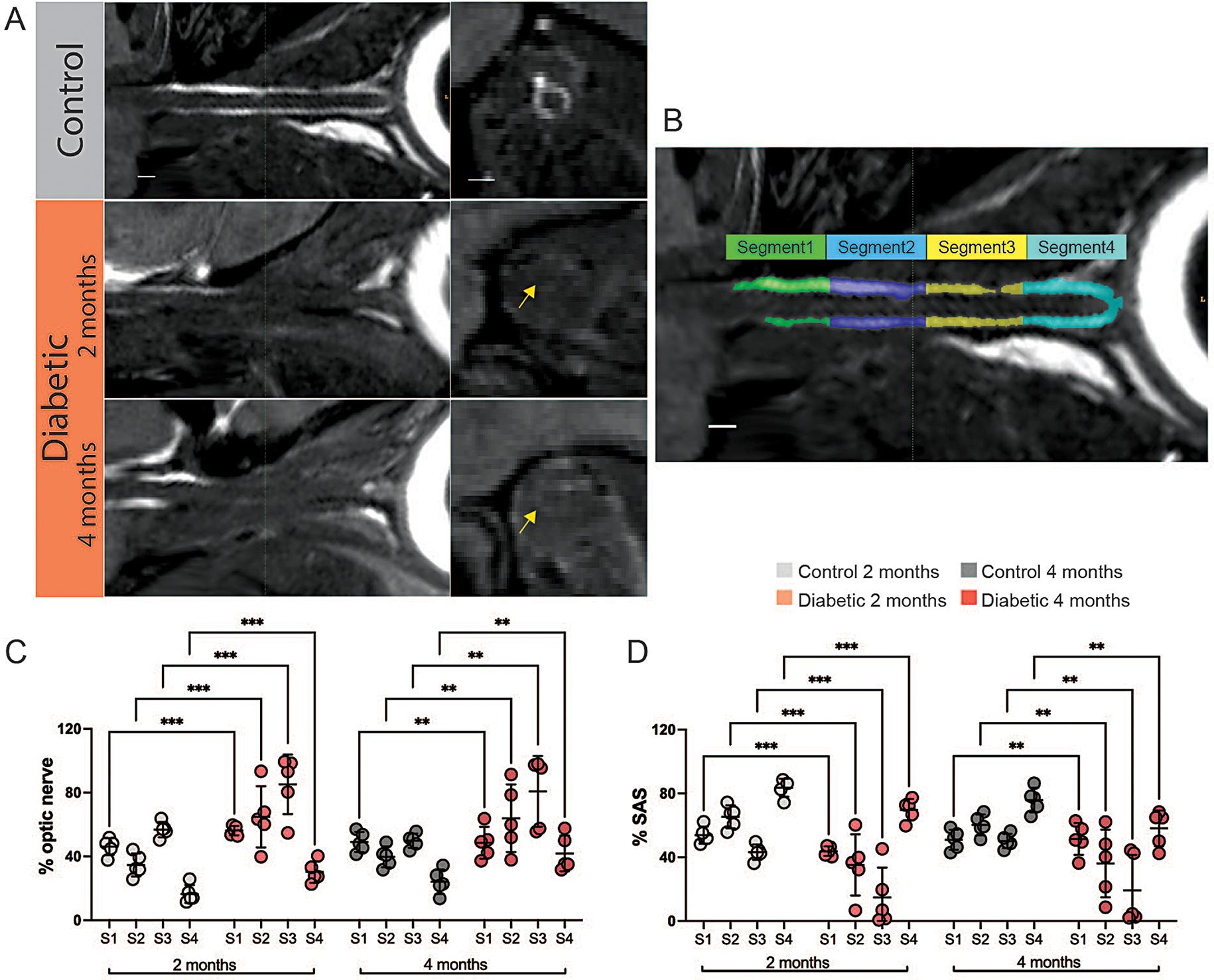
In vivo magnetic resonance imaging revealing reduced CSF space in diabetic optic nerves. (**A**) Representative curved planar reconstruction of the optic nerve of representative control (4 months) and diabetic mice at two- or four-months after sham or STZ injection. Left: Longitudinal view, right: Cross section view. Green line indicate location of represented cross section view. Yellow arrow indicates diminished CSF filled subarachnoid space (SAS) in diabetic optic nerves. Scale bar: 200 μm. (**B**) Segmentation of the optic nerve into four segments of equal length: Segment 1 posterior pre-chiasmatic optic nerve part, segments 2 and 3: middle parts of the optic nerve, segment 4: anterior optic nerve part behind the orbit. C + D) Diagrams show the occupation rate (%) of the optic nerves (**C**) and the CSF-filled SAS (**D**) for each segment. *n* = 5, ***P ≤ 0.001, **P ≤ 0.01 between indicated groups by two-way ANOVA with Tukey’s correction. All graphs show mean ± SD

How the pathological progression of diabetes affects the volume of the optic nerve remains largely unknown. To map the possible effects of diabetes on nerve volume, in vivo non-invasive MRI was next performed on anesthetized mice, followed by a curved planar reconstruction of the optic nerve. The reconstructed optic nerve and surrounding SAS were subdivided into four segments of equal length: one posterior (segment 1), two middle (segments 2 and 3), and one anterior segment (segment 4) (Fig. 4B). The occupation rate for these optic nerve segments and the SAS were then determined (Fig. 4C-D). Strikingly, for two- and four-month diabetic mouse groups, the occupation rate was larger in all optic nerve segments compared to controls (Fig. 4C). Conversely, the occupation rates of the SAS surrounding the optic nerves of diabetic mice were smaller in all segments compared to controls (Fig. 4D). These findings indicate swelling of the optic nerves and compression of the surrounding CSF-filled SAS in the diabetic mice as compared to controls.

### Repeated glucose challenge accelerates ocular glymphatic transport in non-diabetic mice

**Fig. 5:**
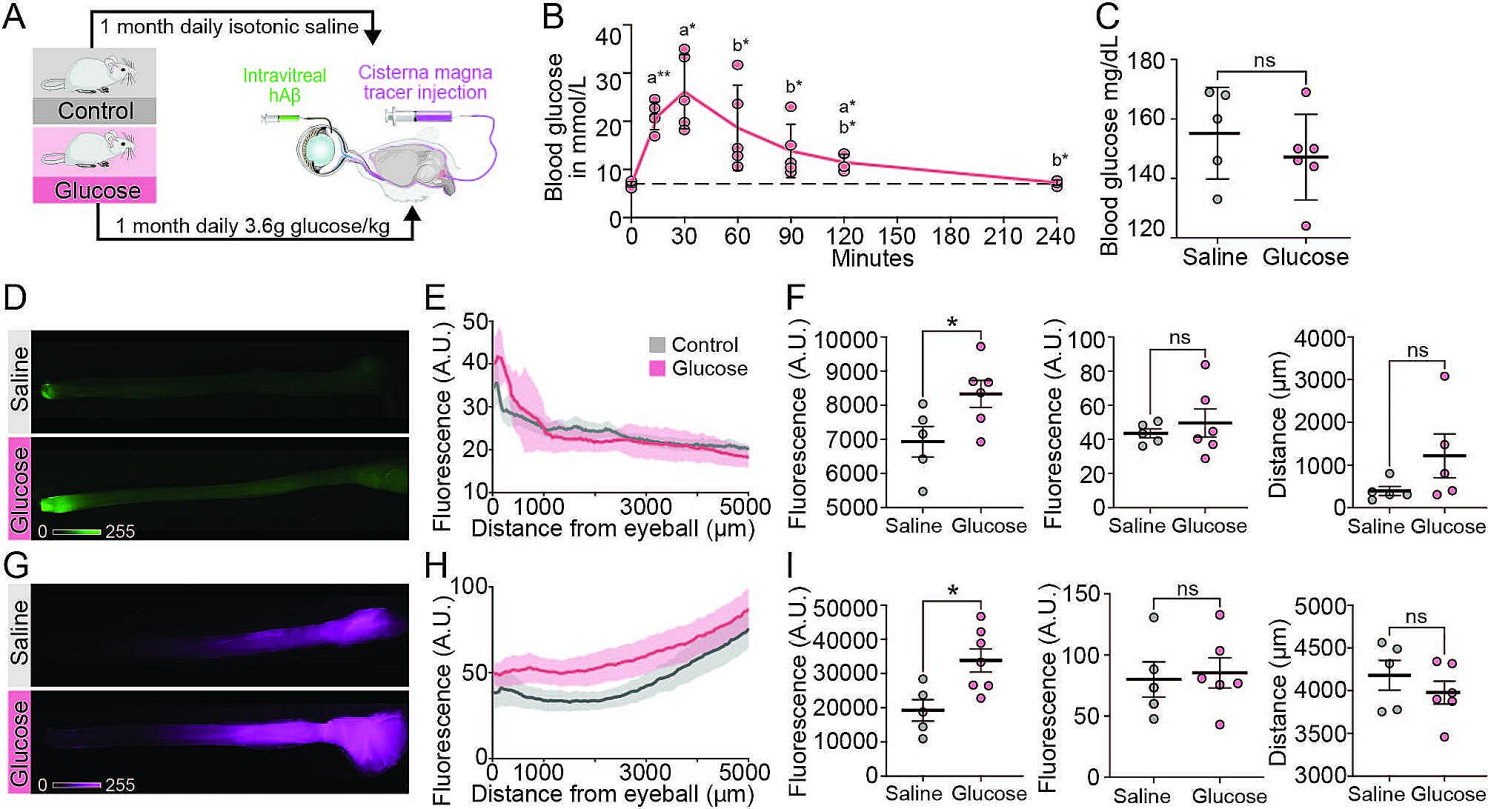
Repeated glucose challenge leads to abnormal glymphatic fluid transport in optic nerve of healthy mice. (A) Schematic of daily (except weekends) intraperitoneal administration of 1 M glucose solution or isotonic saline in healthy mice over a month followed by intravitreal (ITV) and cisterna magna (CM) injection of glymphatic-relevant tracers. (B) Representative plot of transient change of blood glucose levels in healthy awake mice after intraperitoneal injection of glucose (n = 5). One-way ANOVA, repeated measures with Geisser-Greenhouse correction. Significant differences to baseline (t = 0 min) indicated with the letter a. Significant differences to peak value (t = 30 min) indicated by letter b with *P ≤ 0.05. (C) Blood glucose levels of awake mice directly prior to undergoing the final experiment of intravitreal and intracisternal tracer injection (n = 5–6). Unpaired two-tailed t-test with Welch’s correction, ns = P > 0.05. (D-G) Representative macroscopic image of intravitreally (D) and intracisternally (G) injected tracer distributions along the optic nerve. EH) Average total tracer signal intensity over the entire length of the optic nerve (arbitrary units (A.U.), distance (µm) ascending from anterior to posterior) of ITV (E) and CM injected tracer (H). F-I) Total tracer signal intensity of entire optic nerves (left), tracer peak signal (middle), peak distance travelled (µm, ascending from anterior to posterior) (right) for ITV (F) and CM (I) injected tracer (n = 5–7). (F-I) Unpaired two-tailed t-test with Welch’s correction. *P ≤ 0.05 and ns = P > 0.05. All graphs show mean ± SD

The negative results in the diabetic mice with chronic elevations of blood glucose prompted us to test an alternative hypothesis that highly fluctuating blood glucose levels would alter glymphatic fluid transport in the optic nerve. To test this, healthy mice received daily intraperitoneal glucose injections five times every week (excluding the weekends) over one month, thereby inducing one daily transient hyperglycemic episode (Fig. 5A). Control mice were injected with equivalent volumes of isotonic saline. The daily glucose injections induced a temporary elevation of blood glucose levels that peaked around 30 min (average C_max_ 26 mmol/L) and normalized within two hours post injection (Fig. 5B). After one month of regular glucose injections, we investigated ocular glymphatic transport by simultaneous intravitreal and intracisternal tracer injection (Fig. 5A) as described above. To avoid direct effects of hyperglycemia, assessment was performed 72 h after the last glucose injection, a time point when blood glucose levels had normalized in all glucose injected mice (Fig. 5C). Unexpectedly, there was increased total tracer signal in the optic nerves of mice receiving daily glucose injections (Fig. 5D-G), indicating an increased activity of ocular glymphatic transport compared to the control group. Specifically, the glucose mice exhibited elevated total intravitreal tracer signal, representing elevated anterograde ocular glymphatic transport (Fig. 5F, left), while peak signal intensity and peak distance travelled did not significantly differ (Fig. 5F, middle + right). For intracisternally injected tracer, representing retrograde ocular glymphatic transport, the total tracer signal intensity was also significantly elevated (Fig. 5I, left), while the peak signal intensity (Fig. 5I, middle) and peak signal travel distance (Fig. 5I, right) remained comparable in the glucose and saline mouse groups. Thus, anterograde and retrograde ocular glymphatic transport are accelerated in the optic nerve of mice exposed to daily glucose injections.

### Diabetes increases optic nerve vascularization

**Fig. 6:**
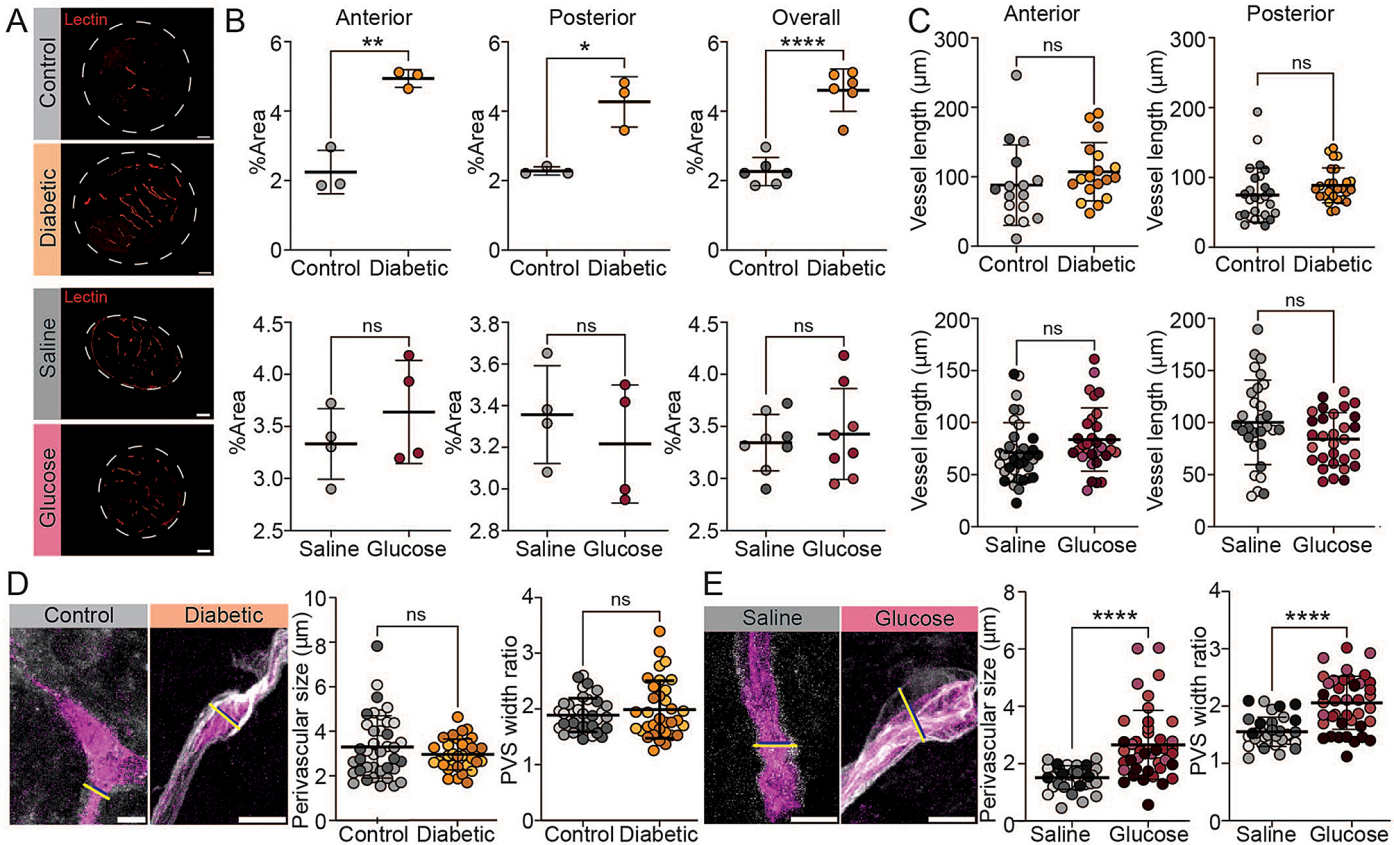
Diabetes increases optic nerve vascularization, while repeated glucose challenge enlarges PVS in the optic nerve. (**A**) Representative images of lectin-stained vasculature in optic nerve cross sections from groups of four-month diabetic and control mice (top) and one-month glucose or saline injected mice (bottom). Scale bar: 50 μm. (**B**) Percentage of lectin-positive stained area in optic nerve cross sections for anterior (first 100 μm behind orbit) (left) and posterior (4–5 mm behind orbit) (right) optic nerve sections (*n* = 3–4 mice, 1 data point = average of 2–4 analyzed cross sections). Right hand graphs represent the summary for anterior and posterior sections. (**C**) Blood vessel length (µm) measured in optic nerve cross sections at different segments (control/diabetic: n(anterior) = 16–18, n(posterior) = 26–27; saline/glucose: n(anterior) = 31–37, n(posterior) = 30). (**D**-**E**) Measurement of perivascular size in optic nerve cross sections of four-month diabetic and control mice (D) and for one-month glucose of saline-injected healthy mice (E), along with representative images (scale bar: 5 μm). Perivascular space size was calculated by subtracting lectin signal diameter (blue line ROIs) from the tracer signal diameter (yellow line ROIs) (left graphs), and width ratio calculated by diving tracer signal diameter with lectin signal diameter, thus normalizing for different vessel sizes (right graphs); (n(control/diabetic) = 34–39, n(saline/glucose) = 35–42; 3–4 different animals). (**C**-**E**) Color gradings indicate single mice. B) Unpaired two-tailed t-test with Welch’s correction; (**C**-**E**) Linear mixed-effects model. ****P ≤ 0.0001, **P ≤ 0.01, ns = *P* > 0.05. All graphs show mean ± SD

Neovascularization is a recognized long-term aspect of diabetic retinopathy alterations in the retina and the optic nerve head region [37, 38], but limited studies address diabetic vascular alterations within the optic nerve itself [39]. Since vascularization can influence glymphatic transport, we assessed the optic nerve vasculature as previously described [8]. Our analysis revealed increased vasculature density in diabetic mice (Fig. 6A, B). In diabetic mice compared to age-matched controls, the total area of lectin-stained optic nerve tissue was about two-fold higher in the anterior (first 100 μm behind the orbit) and posterior segment of the optic nerve (4–5 mm behind the orbit) (Fig. 6A, B). Meanwhile, repeated glucose challenges in healthy mice did not alter the overall vasculature in the optic nerve, as indicated by absent group differences in total lectin signal. In addition, the total vessel length remained comparable between groups (Fig. 6C).

### Repeated glucose challenge in healthy mice enlarges perivascular spaces in the optic nerve

The elevated glymphatic tracer signal in optic nerves of healthy mice receiving repeated glucose challenges occurred in the absence of apparent vascular changes. We next asked whether repeated glucose challenges would expand the perivascular spaces (PVS) and thereby facilitate increased tracer influx. We used a previously described approach to analyze the PVS size [11] of the optic nerve vasculature in diabetic mice and in mice receiving repeated glucose challenges as compared to saline injected control groups. Interestingly, there was PVS expansion in optic nerves of the glucose-treated mice (Fig. 6E), but no such alteration was found in diabetic mice with persistent hyperglycemia (Fig. 6D). As PVS size could differ depending on the vessel size, we also calculated the ratio of PVS size (tracer signal diameter/lectin signal diameter). We detected an increased PVS ratio in the healthy mice with repeated glucose treatments, while the PVS ratio in diabetic mice did not differ from control values. Thus, repeated transient hyperglycemic events expanded the PVS diameter in the optic nerve, whereas a chronic persistent elevation of blood glucose in the STZ model had no such effect.

## Discussion

The optic nerves transmit visual information from the retina via RGC axons that is processed further to enable visual perception. Several lines of evidence documented that the rodent optic nerve (like the brain) houses an active fluid transport system known as the ocular glymphatic pathway, with mediated bidirectional fluid transport [8, 9]. Here, we showed that chronic diabetes did not affect anterograde or retrograde ocular glymphatic influx in the mouse optic nerve (Figs. 1 and 2), despite the development of a severe diabetes phenotype (Suppl. Figure 1), along with classic cellular and vascular retinal hallmarks, including vascular alterations, vascular leakiness to Evan’s Blue, and loss of RGC (Suppl. Figure 3) [6, 7, 40–44]. Further, increased vascularization was evident in the optic nerve of the diabetic mice (Fig. 6A, B) compared to age-matched controls. However, neither anterograde nor retrograde tracer transport along the optic nerve were altered by diabetes with chronic hyperglycemia lasting either two or four months (Fig. 1).

In two experimental glaucoma models, pathologically elevated intraocular pressure (IOP) caused glial lamina disruption, which led to excessive undirected fluid outflow from the retina into the optic nerve [8]. Diabetic mice also exhibited increased IOP compared to controls starting 4.5 weeks after STZ-injection that persisted thereafter (Suppl. Figure 2 A). Although elevated IOP in awake diabetic mice is expected to boost anterograde glymphatic flow [8], ocular glymphatic transport did not differ between anesthetized diabetic and control mice. One caveat here is that ketamine/xylazine anesthesia used during the experimental procedure affects IOP [45], resulting in similar IOP in the diabetic mice and their controls during the glymphatic analysis (Suppl. Figure 2B). It is currently not possible to study ocular glymphatic transport in awake mice, given the invasive nature of tracer injections in the eye. Yet, additional ultrastructural analysis documented that the glial lamina remained intact in diabetic mice (Fig. 3), and that neither the perivascular spaces (Fig. 6) nor the meningeal lymphatic structures surrounding the optic nerve (Suppl. Figure 5) differed between the two groups. Based on this evidence, we conclude that the chronic and constant elevation of blood glucose in the diabetic mice (blood glucose > 25 mmol/L) had no major effects on either anterograde or retrograde ocular glymphatic flow (meaning perivascular space transport) (Figs. 1 and 2). This study focused on the ocular glymphatic pathway in which amyloid-β is transported across the glia lamina by RGC axons [8] however several alternative routes for amyloid-β clearance exists, such as amyloid-β transport across the blood-brain-barrier [46].

Studies of the ocular glymphatic system currently require ex vivo determination of tracer distribution in the optic nerve. Therefore, we performed an additional in vivo noninvasive MRI approach for structural analysis of the optic nerve in our diabetes mouse model without contrast agent injection (Fig. 4). Here, we divided the optic nerves into four equal segments to examine possible volume differences in the segments closest to the inlets of anterograde and retrograde glymphatic fluid influx. Interestingly, our MRI analysis revealed an increased optic nerve occupation rate in all segments of optic nerves in diabetic mice (Fig. 4C), while the SAS space occupation rate was lower than in the control group (Fig. 4D). The usage of a cryogenically cooled probe enabled high resolution structural MRI, which visualized both swelling of the optic nerve and shrinkage of the surrounding CSF-filled SAS. Diffusion tensor imaging has earlier documented decreased diffusivity in optic nerves in STZ-model diabetic rats, possibly reflecting fluid accumulation in the optic nerve [20]. Other studies have shown delayed brain and spinal cord glymphatic clearance in diabetes type II rat models [19, 47]. There might be several reasons why diabetes differentially affects ocular and brain glymphatic transport. First, the sensitivity of ocular glymphatic transport to the chronic elevation of blood glucose may differ from that of brain and spinal cord glymphatics. Second, the swelling of the optic nerve in diabetic mice is expected to increase tissue resistance, which over two to four months could result in creation of alternative low resistance routes that maintain glymphatic flow between the parallel myelinated axonal fibers of the optic nerve. The complex ultrastructure of the brain may prevent such adaptive processes. Third, the ex vivo analysis of glymphatic tracers in optic nerve may not be sufficiently sensitive to detect minor changes in glymphatic flow. Finally, it is possible that glymphatic clearance, rather than influx, is impaired in the optic nerve of diabetes model mice. Unfortunately, ocular glymphatic clearance is not presently amenable to study in rodents, given the requirement to inject tracer directly into the thin optic nerve to map tracer loss unconfounded by tracer influx [48]. In the future, use of larger organisms such as pig might enable this procedure.

Fluid retention in tissues can indicate underlying inflammation [49]. In the present study, our analysis of optic nerve homogenates for content of cytokines, chemokines, and matrix-metalloproteases (MMPs) showed minor changes in the inflammation biomarker profiles of diabetic mice (Suppl. Figure 4); only IL12-p70 was significantly increased in the diabetic optic nerve homogenates at two and four months. Of the examined MMPs, only MMP2 was increased in the four-month diabetic optic nerves. Earlier studies in human diabetes patients showed pronounced inflammatory profiles in aqueous humor, vitreous humor, and retina [50–52]. In contrast, present findings indicate only mild inflammation in optic nerves of diabetic mice, as further supported by our analysis of the meningeal lymphatic vessels surrounding the optic nerve. These lymphatic vessels, which drain the optic nerve, were morphologically similar in diabetic mice and controls, without any sign of hypertrophy or sprouting (Suppl. Figure 5). The lack of structural changes in the optic nerve meningeal lymph vessels in diabetic mice supports the conclusion that chronic hyperglycemia in the STZ model does not notably alter glymphatic influx in the optic nerve. One might plausibly consider that homeostatic adaptive mechanisms normalize glymphatic fluid flow in chronic pathological states. Indeed, it is well documented that repeated hyperglycemic events in poorly controlled diabetes are linked to more rapid progression of diabetes pathologies, specifically in relation to more severe microvasculature damage [6, 24]. Thus, we speculated that repeated hyperglycemic events, rather than persistently increased blood glucose, are a driver for glymphatic pathology in the optic nerve. We tested this conjecture by administering daily intraperitoneal glucose injections to otherwise healthy non-diabetic mice (Fig. 5B). Strikingly, anterograde and retrograde ocular glymphatic transport were both accelerated in the mice receiving daily glucose injections (average Cmax 26 mmol/L) compared to saline injected controls (Fig. 5D, G). The total tracer signal of the entire optic nerve for intravitreally injected tracer in the glucose group was significantly 20% higher than in the control groups (glucose: 8332 ± 964 A.U., control: 6933 ± 1007 A.U., *p* = 0.0457). Similarly, the total signal of intracisternally injected tracer in the glucose group was significantly 70% higher than in the control group (glucose: 33,851 ± 3418 A.U., control: 19,227 ± 3133 A.U., *p* = 0.0104). In addition, the optic nerve PVS were enlarged after one month of daily glucose injections in healthy mice, while the PVS size was unaffected in diabetic mouse optic nerves (Fig. 6D, E). Enlargement of the PVS can enable increased fluid traffic, possibly explaining the accelerated ocular glymphatic transport in the mice receiving repeated glucose injections. Present results suggest that transient changes in plasma osmolality due to fluctuating blood glucose levels induce PVS alterations that result in acceleration of glymphatic flow in the long-term. We have recently reported that a long-term high-fat diet in mice inducing severe obesity and hypertension did not alter brain-wide glymphatic fluid transport [53]. Thus, the glymphatic system may better accommodate chronic rather than repeated transient changes in physiological parameters.

Enlarged PVS have been detected in neurovascular and neurodegenerative diseases such as small vessel disease and dementia [54, 55]. Although previous meta-analysis of several studies did not link diabetes with PVS changes [56], a more recent study indicated that PVS score in the basal ganglia was correlated with diabetic retinopathy severity [57]. Further, a cross-sectional study of elderly individuals with hyperglycemia due to metabolic syndrome found reduced diffusivity along the PVS of the deep medullary vein [58]. Thus, present results indicate that PVS remodeling triggered by fluctuating blood glucose levels facilitates glymphatic transport in the optic nerve. In contrast, the chronic elevation of blood glucose in the diabetic mice without insulin treatment did not alter ocular glymphatic transport.

Studies of the ocular glymphatic system have demonstrated the existence of fluid transport along the optic nerve in young and aged wildtype mice and in experimental models of glaucoma [8–11]. The present study provides insights into optic nerve fluid transport in a model of chronic diabetes and in otherwise healthy wildtype mice experiencing transient increases in plasma glucose. Strikingly, anterograde and retrograde glymphatic transport (tracer experiments, Figs. 1 and 2) both remained unchanged in the diabetic mice. The optic nerve swelling (MRI, Fig. 4) in diabetic mice possibly reflects adaptation to the chronic hyperglycemic state by the creation of novel low-resistance fluid routes within the optic nerve. Such microscopic changes in tissue fluid flow cannot be detected ex vivo after harvesting the optic nerve [59]. In contrast, transient hyperglycemia in mice exposed to repeated glucose challenges showed parallel increases in anterograde and retrograde ocular glymphatic transport, which were linked to structural changes in the PVS. The mechanisms driving these functional and structural changes in mice exposed to repeated glucose challenges call for further investigation. However, we propose that transiently spiking blood glucose levels may cause a significant fluid shift across the vascular wall, thereby imposing structural changes in the PVS. Our study highlights that fluctuating blood glucose levels, or by extension poorly controlled diabetes, is a driving factor in ocular glymphatic dysfunction, which is however able to adapt to the stable hyperglycemia of untreated diabetes.

### Electronic supplementary material

Below is the link to the electronic supplementary material.

Supplementary Figures

Supplementary Methods

## Data Availability

The datasets used and/or analysed during this study are available from the corresponding author upon reasonable request.

## Abbreviations

A.U.: Arbitrary units
AQP4: Aquaporin-4
BSA: Bovine serum albumin
CM: Cisterna magna
CSF: Cerebrospinal fluid
CPR: Curved planar reformation
IOP: Intraocular pressure
MMP: Matrix-metalloproteases
MRI: Magnetic resonance imaging
PBS: Phosphate buffered saline
PVS: Perivascular spaces
RGC: Retinal ganglion cells
SAS: Subarachnoid space
SD: Standard deviation
STZ: Streptozotocin
