# Supplementary material for: Transient but not chronic hyperglycemia accelerates ocular glymphatic transport
Source: Fluids Barriers CNS. 2024 Mar 12;21:26. doi: 10.1186/s12987-024-00524-w (PMC10935920; doi:10.1186/s12987-024-00524-w)
Supplement: Supplementary file 1 — Supplementary Figures [file 12987_2024_524_MOESM1_ESM.docx]

**Supplementary Figures**

**Transient but not chronic hyperglycemia ­­accelerates ocular glymphatic transport**

Christine Delle^1^, Xiaowei Wang^2,3^, Michael Giannetto^2^, Evan Newbold^2^, Weiguo Peng^2^, Ryszard Stefan Gomolka^1^, Antonio Ladrón-de-Guevara^2^, Neža Cankar^1^, Elise Schiøler Nielsen^1^, Celia Kjaerby^1^, Pia Weikop^1^, Yuki Mori^1^, Maiken Nedergaard^1,2*^

^1^Center for Translational Neuromedicine, Faculty of Medical and Health Sciences, University of Copenhagen, Blegdamsvej 3B, 2200 Copenhagen N, Denmark.

^2^Center for Translational Neuromedicine, University of Rochester Medical School, Elmwood Avenue 601, Rochester, NY 14642, USA.

^3^School of Medicine, University of California, San Francisco, 10 Koret Way, San Francisco CA 94117.

^*^ Corresponding author

**
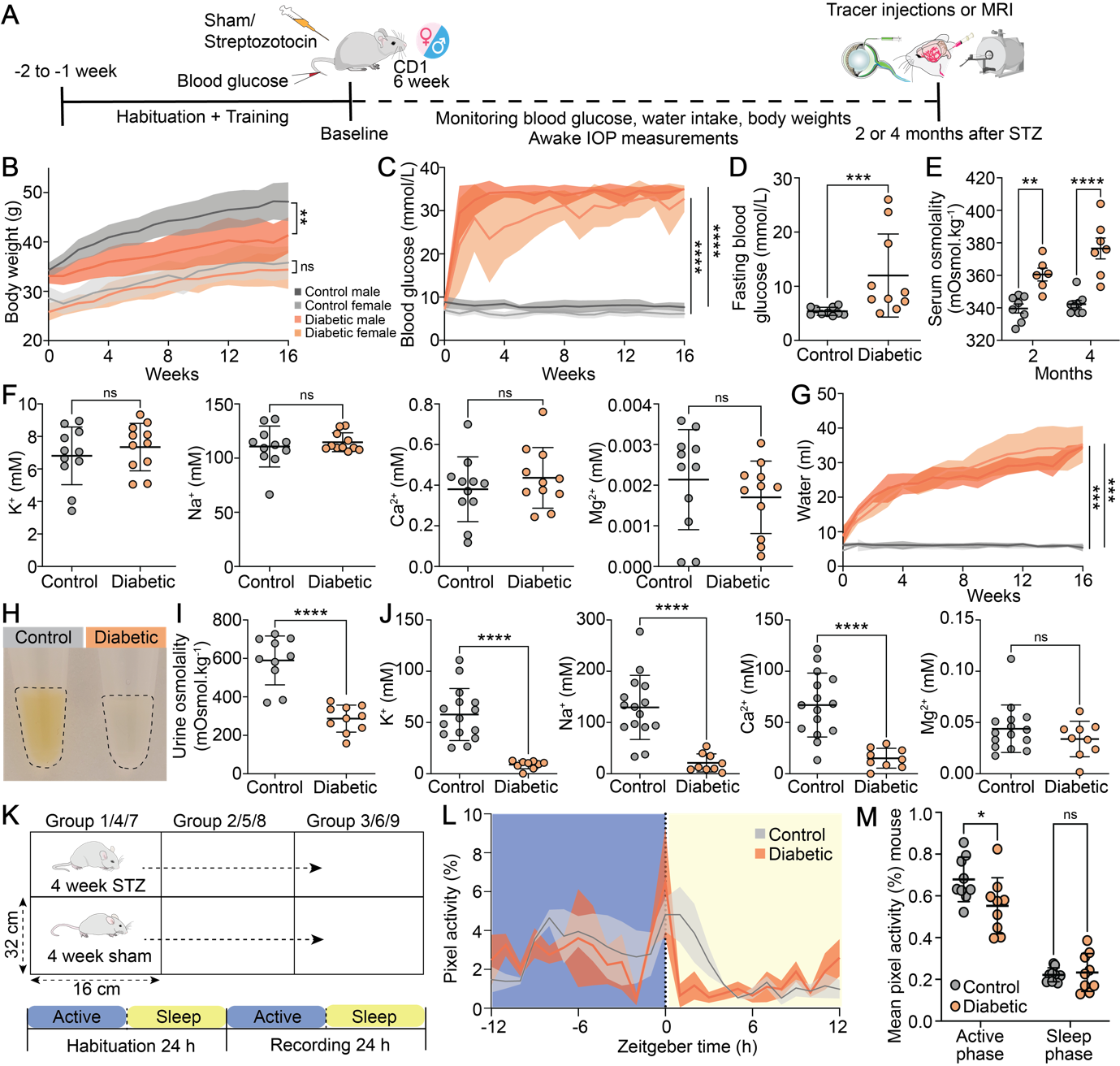
**

**Supplementary Figure 1: Experimental design and basic parameters.** A) Schematic experimental design and timeline of the diabetic study. B) Body weights of control and diabetic mice monitored over four months. n=10, *****P*  ≤ 0.0001 and ns = *P* > 0.05 between indicated groups by two-way ANOVA with Šídák's correction. C) Development of blood glucose levels over the experimental time course. n=10, *****P* ≤ 0.0001 between indicated groups by two-way ANOVA with Šídák's correction. D) Blood glucose levels after 8 h of fasting 2 weeks after diabetes induction. n= 10, ****P* ≤ 0.001, unpaired two-tailed t-test, Mann-Whitney test. E) Blood serum osmolality. n=6-8, *****P* ≤ 0.0001, ***P* ≤ 0.01 between indicated groups by ordinary two-way ANOVA with Tukey’s correction. F) levels of cations (K^+^, Na^+^, Ca^2+^, Mg^2+^) in blood serum obtained from control and diabetic mice at four-month timepoint. n=11, ns = *P* > 0.05, unpaired two-tailed t test with Welch’s correction. G) Water intake average per animal per day over the time course of the study. n=3-6, *****P* ≤ 0.0001 between indicated groups by two-way ANOVA with Šídák's correction. H) Exemplary image of urine color from a four-month diabetic and control mouse. I) Urine osmolality (n=10, *****P* ≤ 0.0001 unpaired two-tailed t-test with Welch’s correction) and J) urine cations (K^+^, Na^+^, Ca^2+^, Mg^2+^) at four months timepoint (n=9-15, *****P* ≤ 0.0001, ns = *P* > 0.05, unpaired two-tailed t-test with Welch’s correction). K) Experimental setup of the activity study. Diabetic or control mice (n = 9 cages with 4-5 mice per cage, four weeks after sham or STZ injection) were habituated for one cycle of active (night, blue colored) and sleep phase (day, yellow colored) (lights on 7am, lights off 7pm) and then recorded for one cycle of active and sleep phase. L) Summary trace of pixel activity of one recorded group of control and diabetic mice during one cycle of active and sleep phase. M) Mean pixel activity in percent per mouse of a total of nine recorded groups of either diabetic or control mice. n=9, **P*  ≤ 0.05 and ns = *P* > 0.05 between indicated groups by two-way ANOVA with Šídák's correction. All graphs show mean ± SD. MRI illustration in A) was created with BioRender.com. Other illustrations by Dan Xue.


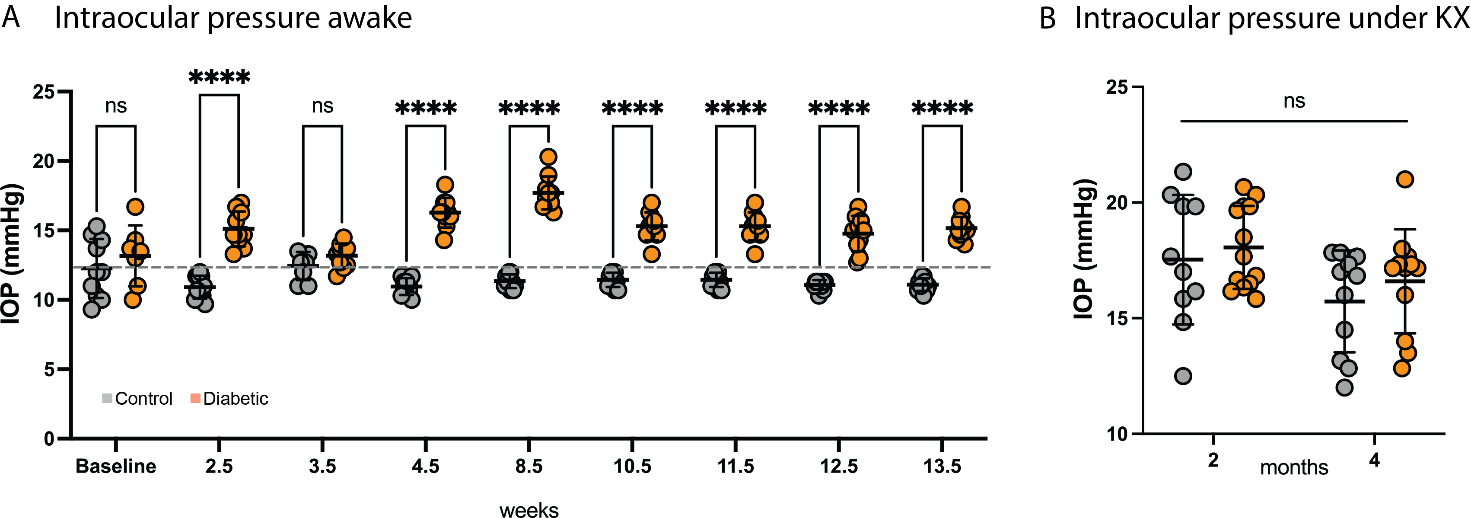


**Supplementary Figure 2: Intraocular pressure of awake and anesthetized mice.** A) Intraocular pressure (IOP) of trained awake mice before STZ or sham injection (baseline) monitored over the time course of diabetes progression up to 13.5 weeks of diabetes. n=7-10, *****P* ≤ 0.0001 and ns = *P* > 0.05 between indicated groups by two-way ANOVA with Šídák's correction. B) IOP of diabetic and control mice at four-month time point after 5 minutes of ketamine (100mg/kg)/xylazine (20mg/kg) (K/X) anesthesia. n=10-13, ns = *P* > 0.05 between indicated groups by ordinary two-way ANOVA with Tukey’s correction. All graphs show mean ± SD.


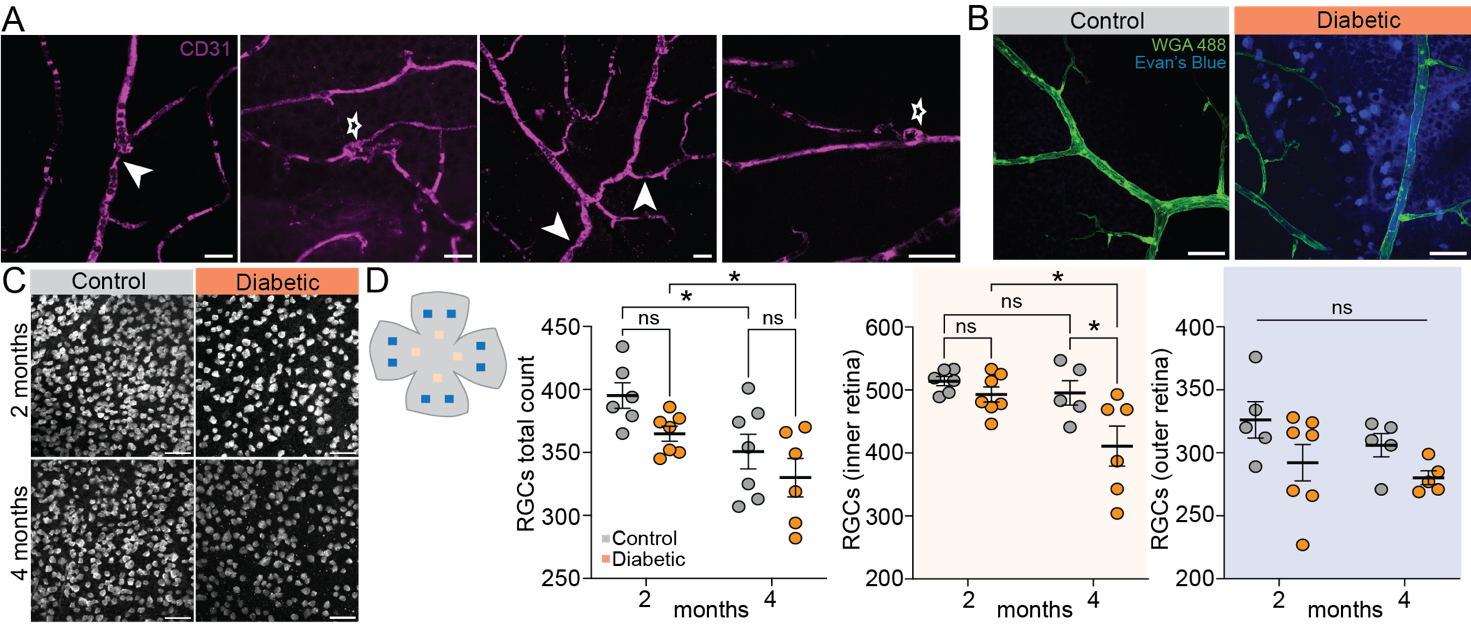


**Supplementary Figure 3: Retinal cellular and vascular pathology.** A) Representative images of retinal vasculature stained for CD31 of four-month diabetic mice. Arrows indicate vascular alterations, stars indicate inflated vascular diameter. Scale bar: 25 μm. B) Test of inner blood retinal barrier integrity in four-month diabetic and age-matched control mice via intravenous Evan’s blue (blue) injection and following lectin-488-PBS cardiac perfusion to label the vasculature (n=2-3). Controls showed no leakage of Evan’s Blue (left), while Evan’s Blue leaked from the retinal vasculature into the periphery (note partial uptake by retinal cells) in diabetic mice (right). Scale bar: 50 μm. C) Representative images of retinal ganglion cells (RGCs) in retinas of control and diabetic mice two and four months after sham or STZ injection. Scale bar: 50 μm. D) Quantification of RGCs for whole retina (left), inner retina (middle) and outer retina (right). n=6-7, **P* ≤ 0.05 and ns = *P* > 0.05 between indicated groups by ordinary two-way ANOVA with Tukey correction. All graphs show mean ± SD.


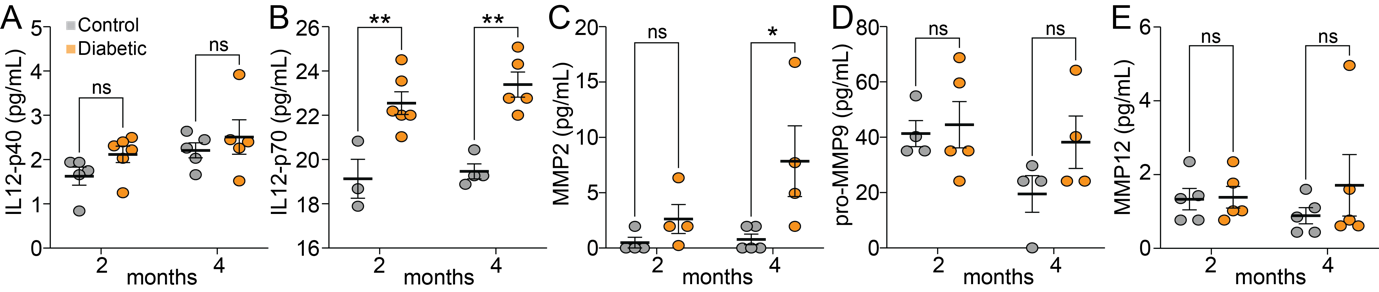


**Supplementary Figure 4: Inflammation of the optic nerve in diabetes.** A-E) Concentration of the cytokines IL-12-p40 (A), IL12-p70 (B) and metalloproteases MMP2 (C), pro-MMP9 (D) and MMP12 (E) in optic nerve homogenates from control and diabetic mice after two or four months of sham or STZ injection. Additional cytokines and chemokines (e.g., TNFα, IL-1α, IL-1β, IL-6, IFNγ, VEGF) not displayed but showed no differences between groups. n=3-6, ***P* ≤ 0.01, **P* ≤ 0.05 and ns = *P* > 0.05 between indicated groups by ordinary two-way ANOVA with Šídák's correction. All graphs show mean ± SD.


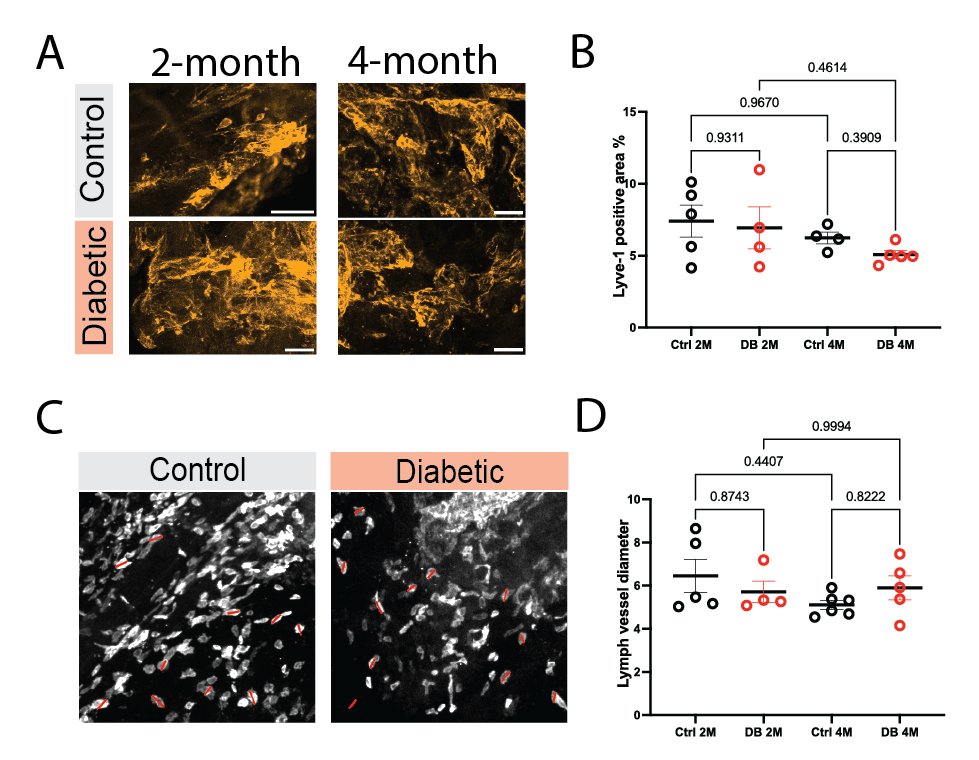


**Supplementary Figure 5: Absence of optic nerve meningeal lymphatic alterations in diabetic mice.** A) Exemplary images of dural lymphatic vasculature of the optic nerve after two and four months of sham (control) or STZ injection (diabetic). B) Total lymphatic Lyve-1 positive area of the optic nerve dura whole mount in percent. n=4-5, displayed *P* values between indicated groups by ordinary two-way ANOVA with Tukey correction. Scale bars 50 µm. C) Example optic nerve dural lymph vessel of control and four months diabetic mouse. D) Average lymph vessel diameters of the optic nerve dura. Lyve-1 signal in white for optimal contrast, exemplary line ROIs in orange. n=4-5 (average values of 10-15 lymph vessels measured per animal), displayed *P* values between indicated groups by ordinary two-way ANOVA with Tukey correction. All graphs show mean ± SD.
