# Supplementary material for: Transient but not chronic hyperglycemia accelerates ocular glymphatic transport
Source: Fluids Barriers CNS. 2024 Mar 12;21:26. doi: 10.1186/s12987-024-00524-w (PMC10935920; doi:10.1186/s12987-024-00524-w)
Supplement: Supplementary file 2 — Supplementary Methods [file 12987_2024_524_MOESM2_ESM.docx]

**Transient but not chronic hyperglycemia accelerates ocular glymphatic transport**

Christine Delle^1^, Xiaowei Wang^2,3^, Michael Giannetto^2^, Evan Newbold^2^, Weiguo Peng^2^, Ryszard Stefan Gomolka^1^, Antonio Ladrón-de-Guevara^2^, Neža Cankar^1^, Elise Schiøler Nielsen^1^, Celia Kjaerby^1^, Pia Weikop^1^, Yuki Mori^1^, Maiken Nedergaard^1,2*^

^1^Center for Translational Neuromedicine, Faculty of Medical and Health Sciences, University of Copenhagen, Blegdamsvej 3B, 2200 Copenhagen N, Denmark.

^2^Center for Translational Neuromedicine, University of Rochester Medical School, Elmwood Avenue 601, Rochester, NY 14642, USA.

^3^School of Medicine, University of California, San Francisco, 10 Koret Way, San Francisco CA 94117.

* Corresponding author

**Supplementary methods: Additional information for the methods utilized to obtain supplemental data presented in supplementary figures.**

**Animal monitoring**

Diabetic mice were monitored daily after streptozotocin (STZ) injection. Standard chow was supplied on the cage floor. Additional wettened food was provided to prevent animals from potential dehydration. General health checks and body weight monitoring were performed daily on diabetic animals according to local animal welfare guidelines. If necessary (weight loss/dehydration) diabetic mice received single subcutaneous injections of sterile isotonic saline. Control animals were weighed weekly.

**Water consumption**

Water bottles were weighed daily before and after refill of control and diabetic mice cages. Water intake per day was calculated as the average consumption per day by randomly chosen three obtained measurements per week and dividing by the number of mice per cage. Water intake was then calculated and plotted per day per mouse.

**Blood glucose measurements**

Non-fasting baseline levels of all mice were obtained prior to start of diabetes induction or repeated glucose injection experiments. For STZ injected mice blood glucose levels were monitored 48 hours post administration to confirm pancreatic β cell toxicity of STZ and thereafter weekly in non-fasted control and diabetic mice. No insulin was administered, and hyperglycemia was not treated for the duration of the experiments. Fasting blood glucose was measured two weeks after STZ or sham injection after fasting mice for 8 hours in fresh cages.

In mice receiving daily glucose or saline injections non-fasting blood glucose levels were monitored weekly and directly before undergoing final tracer injection experiments.

All blood glucose measurements were conducted using a BAYER Countour apparatus and test stripes (Ascensia).

**Blood and urine sampling**

Blood samples were collected in form of trunk blood after MRI imaging and decapitation of mice. Otherwise, a blood sample was drawn by cardiac puncture prior to perfusion. Blood was collected in sterile 1.5 mL centrifuge tubes. At room temperature samples were allowed to coagulate for approx. 20 minutes and then spun down (2,000 x g at 4°C for 10 minutes). Supernatants were pipetted into fresh tubes and immediately snap frozen on dry ice and stored until further use at -20°C.

For urine sampling mice were picked up as for an intraperitoneal injection and samples obtained from urinating mice. Otherwise, after terminal procedure (MRI or tracer injection) urine was immediately collected via bladder puncture with a sterile syringe connected to a 30G needle. Urine was collected in sterile and pyrogen free 1.5mL centrifuge tubes and immediately stored on ice prior to brief centrifugation (4 minutes, 10.000 x G, 4°C). Supernatants were transferred into fresh, sterile tubes and stored until analysis at -20°C for maximum two weeks.

**Osmolality**

Osmolality of blood serum and urine samples was measured using a Micro-Osmometer Automatic Type 15/15 M (Löser Messtechnik, Berlin, Germany) according to manufacturer’s protocol. Per sample 100μl of sample was measured after calibration with standard solution (Löser Messtechnik, Berlin, Germany).

**Ion detection**

For ion measurements samples were prepared as followed: equal amounts (e.g., 50μL + 50μL) of urine and acetonitrile (ACN) (Merck KGaA, Darmstadt, Germany, purchase from SigmaAldrich, Ref. 1.00029.2500) were mixed to precipitate proteins. For serum samples equal volume were precipitated with equal volume of ACN. After brief mixing, samples were spun down at maximum speed (14.8 rpm) for 10 minutes at 4°C. Supernatants were transferred into fresh tubes and once again spun down as described before to remove any remaining precipitates. 5 μL of supernatant was then given into 695μL of MilliQ water in a chromatography tube. Ion concentration in samples was measured using a cation chromatography system (Dionex Aquion IC 1100 Detector Thermo system (Fisher Scientific Inc., US), equipped with a CS12A 4 mm analytical (Fisher Scientific Inc., US) and a Dionex IonPac CG12A 4 mm guard column set (Fisher Scientific Inc., US). Samples were eluted for 20 minutes with isocratic 20 mmol/L methanesulfonic acid (MSA) with a flow rate of 0.36mL/min. Ion concentrations for potassium, sodium, calcium, and magnesium were measured by elution time and strength of measured current and calculated from a validated in-house standard curve. Unknown sample ion concentrations were determined based on the linear fit and then back calculated taking the dilution factor into account. The computerized data acquisition system is a Thermo Scientific™ Chromeleon™ Chromatography Data System (CDS) software.

**Activity tracking**

The activity of diabetic and control mice was monitored after 4 weeks of STZ or sham injection respectively. Therefore, home cages of mice were transferred to a behavioral arena, controlled in the same manner as the housing stable, and lids and grids removed. High non-transparent Plexiglas walls separated the cages. Per round, 3 cages of diabetic and 3 cages of control mice with a total cage consisting of 4-5 mice were monitored. The activity study was conducted on a total of 9 cages per group. After a 24 h habituation phase (1 cycle of active and sleep phase) groups were videorecorded for 24 h to monitor one cycle of active and one cycle of sleep phase. Recording was acquired using Synapse (Tucker Davis Technologies) and Basler cameras. Videos were tracked using EthoVision XT and activity (%) was calculated and plotted in mean pixel activity per mouse per group by dividing the readout of mean pixel activity by the total number of mice in each group.

**Intraocular pressure**

Mice were trained daily for one week prior to obtaining weekly intraocular pressure (IOP) data on awake animals. Mice were placed in a body strainer similar as previously described procedure ^1^ and eye pressure measured with a handheld tonometer (TonoLab, icare Finland Oy). This allowed fast accurate measurements without application of local anesthesia and was without irritation for the animal. IOP measurements prior to intravitreal tracer injections were conducted in anesthetized animals exactly five minutes after intraperitoneal injection of anesthesia (ketamine/xylazine; 100 mg/kg and 20 mg/kg respectively). Per eye three 6-sequential measurements were conducted which were averaged to a single IOP readout per mouse per timepoint similar as previously described ^2^.

**Staining and imaging for retinal ganglion cells (RGCs)**

To stain for RGCs, enucleated eyes were incubated overnight at 4°C with rabbit anti-RNA binding protein with multiple splicing (RBPMS) (1:250; GeneTex) and detected with secondary antibody (1:500) for 2h at RT. Cy3-conjugated donkey anti-rabbit secondary antibody (1:500; Molecular Probes/Jackson ImmunoResearch). After staining retina whole mounts were mounted. RGCs confocal images were acquired by a Nikon Eclipse Ti2 microscope with a Plan Apo x40/1.40 numerical aperture (NA) oil objective controlled by an imaging software NIS-Elements AR 4.50.00.

**Cytokine and metalloprotease assay of optic nerve homogenates**

Optic nerves were harvested after decapitation of anesthetized mice 2 and 4 months after sham injection or diabetes onset. Tissue preparation was conducted similar as previously described ^3^. In brief, optic nerves were immediately snap frozen in sterile centrifuge tubes on dry ice and stored at -80°C until preparation of homogenates. For homogenization optic nerves were transferred into a 0.5mL containing ceramic beats (Precellys Lysing Kit, P000933-LYSKO-A, Bertin Technologies, France) and 200 mL of a protease inhibitor solution (P2714-1BTL, Sigma-Aldrich, 1:100 in PBS). Utilizing a Precellys homogenizer (Precellys Evolution, Bertin Technologies, France) samples were homogenized at 500 rpm for 20 seconds repeated for three times with 30 seconds breaks. Immediately after, tissue debris were pelleted by centrifugation for 10 minutes at 10.000 x G at 4°C. Supernatants were transferred into sterile and pyrogen free 0.5 mL centrifuge tubes. A BCA assay (BCA protein assay kit, ab207002, Abcam) was performed according to the manufacturer’s protocol to determine the protein contents. All samples were then adjusted to an equal protein concentration of 180 μg/mL in a total volume of 125μL. Samples were immediately snap frozen on dry ice and stored at -80°C until analysis. Cytokines and matrix metalloproteases were analyzed utilizing the Multiplex Laser Bead platform (MD32 and MDMM-P,O) by Eve Technologies (Alberta, Canada).

**Staining for meningeal lymphatic vessels**

To detect lymphatic vessels in the meningeal sheath of the optic nerve a whole mount meningeal sheath was prepared by carefully pulling off from the anterior site of the optic nerve and fixating the optic nerve on the posterior position. The meningeal sheath was then incubated with rat anti-LYVE1 antibody (1:250, eBioscience) overnight at 4°C. The primary antibodies were detected with Alexa Fluor 594-conjugated goat anti-rat secondary antibody (1:500, Abcam, AB175732). After two hours of secondary antibody incubation at room temperature wi­­­­th mild shaking, meningeal sheats were washed three times with PBS at room temperature for 5–10 minutes each before mounting between two cover slides. Dural confocal images were acquired with a Nikon Eclipse Ti2 microscope with a Plan NOO Apo x10/1.40 numerical aperture (NA) objective controlled by an imaging software NIS-Elements AR 4.50.00.

The total LYVE1 positive area was expressed as percental area of the total dura wholemount tissue, using ImageJ (version 2.1.0/1.53c). The diameter of lymph vessels was measured by drawing a line ROI across well-defined lymph vessels with sharp demarcation from surrounding tissue or background signal.

**Vascular labeling**

A subset of mice underwent vascular labeling with wheat germ agglutinin (WGA) (lectin) to assess optic nerve vascularization. Mice were briefly perfused with lectin diluted in PBS. For this 300 µL of Alexa Fluor 555 or 647 conjugated lectin (W32464 or W32466, Thermo Fisher Scientific) in a concentration of 1mg/mL were diluted in 20 mL of 1x PBS and mice perfused for five minutes. Thereafter optic nerves and eyes were immediately harvested.

**Tracer analysis in retinal whole mounts**

Retinal whole mount images were analyzed using ImageJ (version 2.1.0/1.53c) by drawing multiple line ROIs (10-20) from the center (location of optic nerve head) to the utmost segment of the retina. An in-house computed macro was used to transfer line ROI readout into a csv file further used in a Python code (version 3.7+) computing average AUC for the entire trace and segmented distances.

**Electron microscopy**

Fully anaesthetized mice were perfused with 2% glutaraldehyde in 0.05M phosphatebuffer (pH 7.2). Then eyes with attached optic nerves were carefully removed and fixed in the perfusion solution for another hour at room temperature. Thereafter, specimens were rinsed 3x in 0.15 M sodium phosphate buffer (pH 7.2), and postfixed in 1% w/v OsO4 with 0.05M K3Fe(CN)6 in 0.12 M sodium phosphate buffer (pH 7.2) for two hours. The specimens were dehydrated in graded series of ethanol, transferred to propylene oxide and embedded in Epon according to TEM standard procedures.

Both longitudinal and cross overview sections of the whole structure, 1 µm thick and stained with Toloudine Blue, were prepared to determine the location of the glial lamina. Then thin sections (60 nm) were prepared with a Ultracut UC7 (Leica, Vienna, Austria) and collected on copper grids with Formvar supporting membranes, stained with uranyl acetate and lead citrate, and subsequently examined with a Philips CM 100 Transmission EM (Philips, Eindhoven, Netherlands), operated at an accelerating voltage of 80 kV. Digital images were recorded with an OSIS Veleta digital slow scan 2k x 2k CCD camera and the ITEM software package.

**References:**

1. Icare Finland. Icare LAB tonometer TonoLab. *https://www.youtube.com/watch?v=6Dx45Q5Ue3Y* (2011).

2. Wang, W.-H., Millar, J. C., Pang, I.-H., Wax, M. B. & Clark, A. F. Noninvasive Measurement of Rodent Intraocular Pressure with a Rebound Tonometer. *Invest Ophthalmol Vis Sci* **46**, 4617–4621 (2005).

3. Manouchehrian, O., Ramos, M., Bachiller, S., Lundgaard, I. & Deierborg, T. Acute systemic LPS-exposure impairs perivascular CSF distribution in mice. *J Neuroinflammation* **18**, 34 (2021).
